# Workplace Mental Health Programs for Healthcare Workers: A Systematic Review

**DOI:** 10.3390/ijerph23081002

**Published:** 2026-07-30

**Authors:** Jimmy Jacob, Priyanka Malaiyappan, Vettrivel Arul, Taranjot Kaur, Shanthini Vijayaraman, Greety Antony, Kaviya Ramesh, Maria R. Dasari, Balasankar Ganesan

**Affiliations:** 1 Department of People and Culture, Institute of Health and Management, Melbourne 3051, Australia; jimmy@healthcareers.edu.au; 2 Department of Research and Development, Institute of Health and Management, Melbourne 3051, Australia; priyanka@ihm.edu.au; 3 Department of Community Medicine, Research Methodology and Biostatistics, Vinayaka Mission’s Research Foundation (DU), Salem 636308, India; veldoc4565@gmail.com; 4 School of Nursing, Institute of Health and Management, Melbourne 3051, Australia; taranjot@ihm.edu.au (T.K.); greetyantony@ihm.edu.au (G.A.); 5 Department of Homoeopathic Repertory and Case Taking, Vinayaka Mission’s Research Foundation (DU), Salem 636308, India; shanthini1604@gmail.com (S.V.); drkaviyaramesh1998@gmail.com (K.R.); 6 School of Social Work, Institute of Health and Management, Melbourne 3000, Australia; maria.d@ihm.edu.au; 7 School of Public Health, Institute of Health and Management, Melbourne 3051, Australia; 8 School of Engineering, Swinburne University of Technology, Melbourne 3122, Australia; 9 Global Partner Affiliate Faculty, Arizona State University, Tempe, AZ 85281, USA

**Keywords:** healthcare workers, occupational stress, workplace mental health, burnout, psychological distress

## Abstract

**Highlights:**

**Public health relevance—How does this work relate to a public health issue?**

- Burnout and psychological distress among healthcare workers represent a major public health challenge affecting workforce sustainability and quality of care.
- This review examines the effectiveness of workplace mental health programs, comparing individual and organizational interventions across diverse healthcare settings.

**Public health significance—Why is this work of significance to public health?**

- Individual-level interventions, particularly mindfulness, positive psychology, and digital therapies, show consistent effectiveness in reducing burnout and distress.
- In contrast, organizational interventions remain under-researched, with limited high-quality evidence despite strong observational links to structural determinants, such as workload and trust.

**Public health implications—What are the key implications or messages for practitioners, policy makers and/or researchers in public health?**

- Digital mental health programs provide scalable and accessible solutions without compromising effectiveness in reducing burnout.
- Future research should prioritise rigorous RCTs of organisational interventions (e.g., workload reduction and participatory leadership), as relying solely on individual-level interventions may overlook the structural causes of burnout.

**Abstract:**

Burnout and psychological distress among healthcare workers (HCWs) have become a major workforce challenge, raising an important question: should responsibility for worker wellbeing rest primarily with individuals or with the organization of work itself? This systematic review evaluated workplace mental health programs for HCWs, comparing individual-level psychological interventions with organizational-level approaches. Following PRISMA 2020 guidelines, 54 studies (2016–2026) from more than 18 countries were identified from approximately 7144 records through a PubMed/MEDLINE search. The included studies comprised 29 individually randomized controlled trials (RCTs), 11 cluster-randomized or stepped-wedge trials, and 14 additional non-randomized or contextual studies. ROBINS-I was applied only to the five eligible non-randomized intervention studies—the remaining non-randomized studies contributed contextual evidence and were not included in the ROBINS-I assessment. Here, 41 studies contributed effectiveness evidence, of which 34 (83%) reported a statistically significant improvement in at least 1 primary or key outcome, while 13 studies provided contextual, feasibility, or implementation evidence. Individual-level interventions dominated the evidence base (39/54; 72%). In contrast, organizational interventions were less frequently evaluated, rarely randomized, and showed mixed findings. Observational studies consistently identified workload, organizational trust, and other structural stressors as factors associated with burnout, although causal inferences remain limited. Risk of bias was substantial, with only 7 of 40 randomized trials (17.5%) rated as low risk. All five non-randomized intervention studies assessed using ROBINS-I were judged to have serious risk of bias. Overall, individual-level interventions were evaluated more frequently than organizational interventions and generally reported improvements in mental health outcomes. However, the evidence base was limited by the single-database search, variability in study quality, and methodological limitations. Further well-designed and adequately powered studies, particularly of organizational interventions, are needed to strengthen the evidence base.

## 1. Background

Burnout amongst healthcare workers (HCWs) has reached epidemic proportions. Burnout rates among US physicians and nurses currently report between 35% and 54%, while global estimates vary from 25% to >60% based on cadre, specialty, and the instrument used [1,2]. These rates increased considerably due to the COVID-19 pandemic: prospective cohort studies in several countries reported 60–70% burnout rates among frontline HCWs at times of peak demand, sometimes lasting over a year after the initial phase of the pandemic [3,4]. Using the Maslach definition, burnout manifests as emotional exhaustion and depersonalization; far from being a private failing of individual workers, it is causally linked to medical error, diminished quality of care, intention to quit, and absenteeism, and ultimately to impaired health system performance and worse patient outcomes [5,6,7].

This framing matters for how the problem is addressed. The World Health Organization’s 2022 guidelines on mental health at work identify healthcare as a high-risk sector requiring both individual and organizational interventions [8]. The distinction is consequential. Individual-level programs, such as mindfulness, cognitive-behavioral therapy (CBT), resilience training, and positive psychology, locate the work of coping with stressors within the worker, asking HCWs to build personal capacity to withstand demanding conditions. Organizational interventions, by contrast, target the structural drivers of burnout that originate in the labor process itself: excessive workload, understaffing, poor scheduling, deficient leadership, and moral injury [9]. The WHO position is that effective workplace strategy must combine the two. Yet the research literature has concentrated overwhelmingly on individual-level interventions, in part because they lend themselves more readily to randomized evaluation than do changes to the organization of work.

This asymmetry is itself a sociological phenomenon worth examining. When the evidence base is dominated by interventions that ask workers to adapt to conditions rather than interventions that change those conditions, this may contribute to the individualization of a problem that also has collective and organizational determinants. Previous systematic reviews have not been positioned to surface this tension: they have focused narrowly on mindfulness [10], nurses only [11], or single settings [12]. No recent review has simultaneously compared individual and organizational programs, examined delivery modalities (in-person, digital, and blended), attended to equity dimensions (cadre, sex, shift work, and high-intensity units), and applied RoB 2 and ROBINS-I across experimental and observational designs using a WHO-aligned taxonomy.

This review addresses that gap. We aimed to (1) evaluate the effectiveness of individual-level psychological programs and organizational-level interventions on HCW burnout and psychological distress, (2) compare intervention types using a WHO-aligned three-category taxonomy, (3) assess the role of delivery mode in moderating effectiveness, (4) characterize implementation evidence, and (5) appraise risk of bias and certainty of evidence. Throughout, we treat the balance of evidence between individual and organizational approaches not as a neutral fact but as a question about where the locus of responsibility for healthy work is placed.

## 2. Methods

This systematic review was conducted and reported in accordance with PRISMA 2020 guidelines [13]. The review protocol was not registered in PROSPERO or in any other prospective registry.

### 2.1. Eligibility Criteria

Studies were included if they met the following PICOS criteria. Population: healthcare workers aged 18 years and over—physicians, nurses, allied health professionals, clinical support staff, community health workers, and mixed HCW samples in hospitals, primary care, or long-term care settings. Intervention: workplace-delivered or workplace-enabled mental health programs classified using a WHO-aligned three-category taxonomy [8]: (A) individual-level psychological programs (mindfulness, MBSR, CBT, ACT, resilience training, positive psychology, coaching, relaxation, yoga, and digital psychological apps), (B) organizational-level changes (workload/staffing redesign, scheduling interventions, leadership training, workflow/process change, and team communication change), and (C) multi-component bundles combining A and B. Comparator: usual care, waitlist control, attention control, and alternative program modality. Outcomes: primary: burnout (MBI, CBI, OLBI or derivatives, and emotional exhaustion as a key subscale) and psychological distress (PHQ-9, GAD-7, DASS-21, PSS, and general distress composites); secondary: resilience, wellbeing, presenteeism, work engagement, job satisfaction, turnover intention, absenteeism, and patient safety outcomes. Study design: any follow-up duration, and any healthcare setting. Designs: RCTs, cluster RCTs, quasi-experimental (controlled before–after and stepped-wedge), and observational cohorts with comparator. Published 2016 onward. Uncontrolled pre–post studies, trial protocols, qualitative studies, and previously published syntheses were not eligible as sources of effectiveness evidence. Where such a record described an intervention or workforce population directly relevant to the review question, it was retained solely as a source of contextual, implementation, or feasibility evidence, and was excluded from all effectiveness analyses and comparisons. One systematic review and one trial protocol were retained on this basis—all other review and protocol records were excluded at full-text screening.

### 2.2. Search Strategy

We conducted a systematic search of PubMed/MEDLINE using a series of complementary terms around population (healthcare workers, including nurses, physicians, medical staff, and hospital workforce), outcomes (burnout, mental exhaustion, psychological stress, and workplace stress), interventions (mental health program, resilience training, mindfulness, workplace intervention, and stress management), and study designs (randomized, quasi-experimental controlled trial). This search was from 2016 to March 2026 and yielded about 7144 records. The review was limited to studies published from 2016 onward because this period follows the landmark synthesis by West et al. [7], reflects the contemporary evidence base with the rapid expansion of digital and workplace-delivered interventions, and encompasses the COVID-19 pandemic, during which research on healthcare worker mental health interventions increased substantially. The complete search strategy is provided in Supplementary Table S1.

### 2.3. Intervention Taxonomy and Data Extraction

Interventions were classified using the three-tier WHO-aligned system (individual, organizational, and bundle). Extracted data included cadre (nurse, physician, and allied health), baseline burnout or distress level, sample size and attrition, program components (mindfulness, CBT, resilience training, staffing and scheduling change, and manager training), delivery mode (face-to-face, online/digital, and hybrid), and dose (number of sessions, total contact hours, and program duration). We recorded implementation facilitators (protected time and leadership support), comparator type and intensity, instruments used to assess burnout and distress, and timepoints with standard deviations, standard errors, or confidence intervals. Secondary work outcomes, including turnover intention and absenteeism, were captured where reported. Equity and protected characteristics, sex/gender, shift-work status, assignment to high-intensity units, and contract type, were extracted to support an equity-informed reading of the labor process.

#### Study Selection and Screening

Records retrieved from PubMed/MEDLINE were imported into Rayyan (Rayyan Systems Inc.) for de-duplication and screening. Two independent reviewers screened all records in duplicate using blinded title/abstract and full-text review against the predefined PICOS criteria. Disagreements were resolved by discussion until consensus was reached. All inclusion and exclusion decisions were made by the reviewers. Data were extracted using a piloted spreadsheet, and the complete extraction dataset and risk-of-bias assessments for all included studies are provided as Supplementary Tables S2 and S4. All extracted data were independently rechecked against the original publications. Effect estimates, *p*-values, sample characteristics, intervention details, outcome measures, and study classifications were verified for all included studies. Risk-of-bias assessments, Table 1, figures, and narrative descriptions were updated where necessary to ensure complete consistency.

### 2.4. Risk-of-Bias Assessment

RoB 2 was used for 29 individually randomized trials and Cluster RoB 2 for 11 cluster-randomized or stepped-wedge trials. ROBINS-I was applied only to the five eligible non-randomized intervention studies. Non-randomized studies that did not evaluate intervention effects (e.g., observational association studies), as well as qualitative studies, protocol papers, and systematic reviews, were retained as contextual evidence but were not assessed using ROBINS-I, consistent with its intended application to non-randomized studies of interventions. Particular attention was paid to deviations from intended intervention and missing outcome data, reflecting the high attrition typical of trials embedded in busy clinical workplaces. Self-reported burnout measurement was consistently flagged as a potential source of measurement bias across studies. Because RoB 2 and ROBINS-I use different rating scales, judgements for randomized and non-randomized intervention studies are reported separately and are not pooled into a single distribution (see Section 3.5).

### 2.5. Data Synthesis

Formal meta-analysis was not undertaken, given substantial heterogeneity in burnout instruments (MBI-HSS, MBI-GS, MBI 2-item, CBI, OLBI, and single-item proxies), outcome subscales, follow-up periods, intervention components, and study populations. We conducted a narrative synthesis following the Synthesis Without Meta-analysis (SWiM) guideline [14] organized by taxonomy category and program component. Where available, effect-size metrics (Cohen’s d, standardized mean differences, and within-group change) were extracted or calculated from study-level summary data and presented individually to support qualitative appraisal of intervention effects without quantitative pooling. Certainty of evidence was assessed using a GRADE-informed approach. For each intervention–outcome pair, certainty was evaluated based on risk of bias (RoB 2, Cluster RoB 2, or ROBINS-I), inconsistency, indirectness, imprecision, and potential publication bias. Evidence from randomized studies started at high certainty and evidence from non-randomized studies at low certainty, with downgrading applied where appropriate. Final certainty ratings and reasons for downgrading are reported in Table 2.

## 3. Results

### 3.1. Study Selection

**Figure 1:**
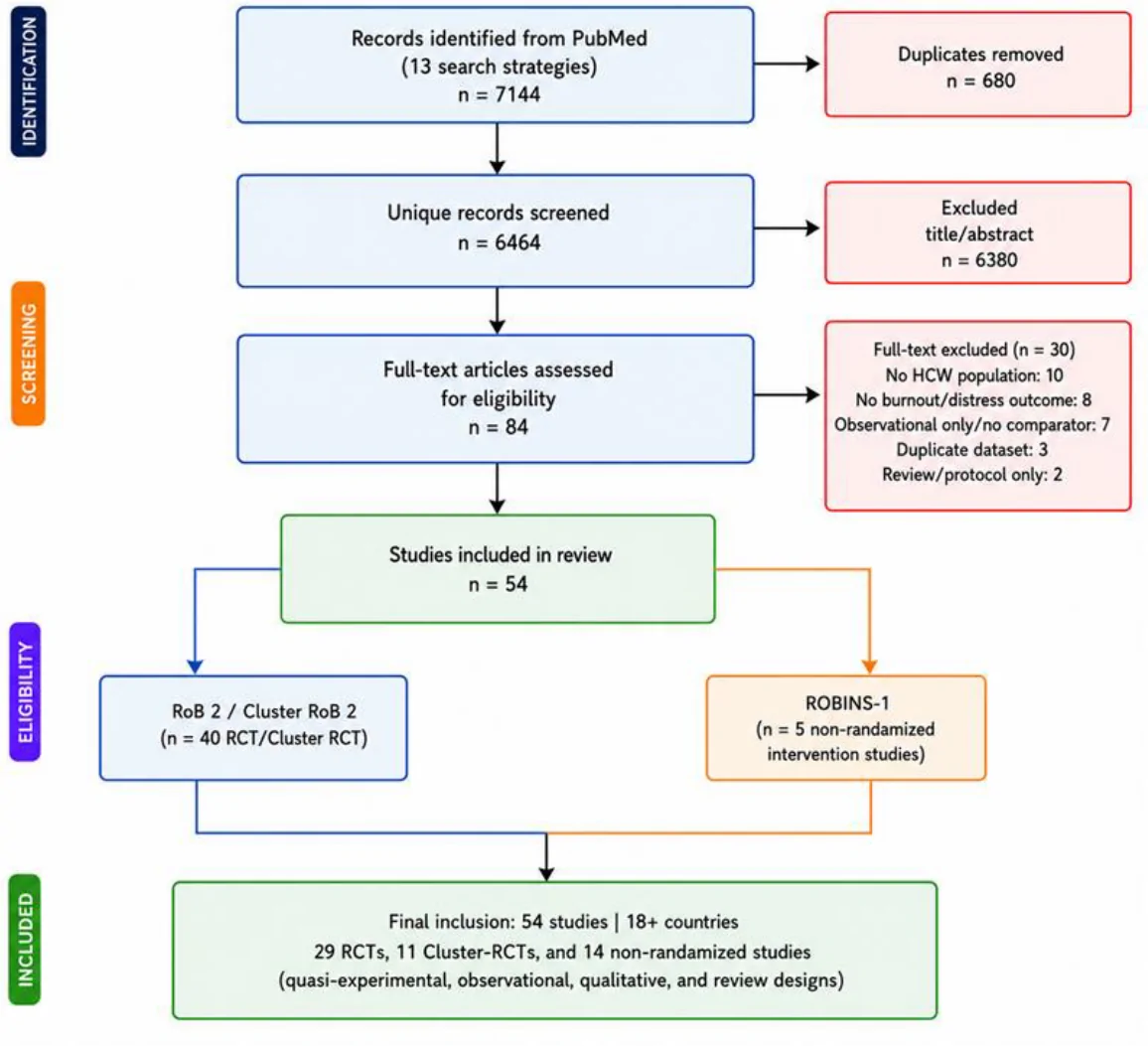
PRISMA 2020 flow diagram of study selection.

This systematic search of PubMed/MEDLINE identified 7144 records. After removing approximately 680 duplicate records, 6464 unique records underwent title and abstract screening, of which approximately 6380 were excluded. A total of 84 full-text articles were assessed for eligibility, and 30 were excluded for the following reasons: no healthcare worker (HCW) population (*n* = 10), no burnout or psychological distress outcome (*n* = 8), observational study without a comparator (*n* = 7), duplicate dataset (*n* = 3), and review or protocol paper (*n* = 2). The final review included 54 studies (Figure 1). The completed PRISMA 2020 checklist is provided in Supplementary Table S3.

### 3.2. Study Characteristics

#### Geographic and Temporal Distribution

**Figure 2:**
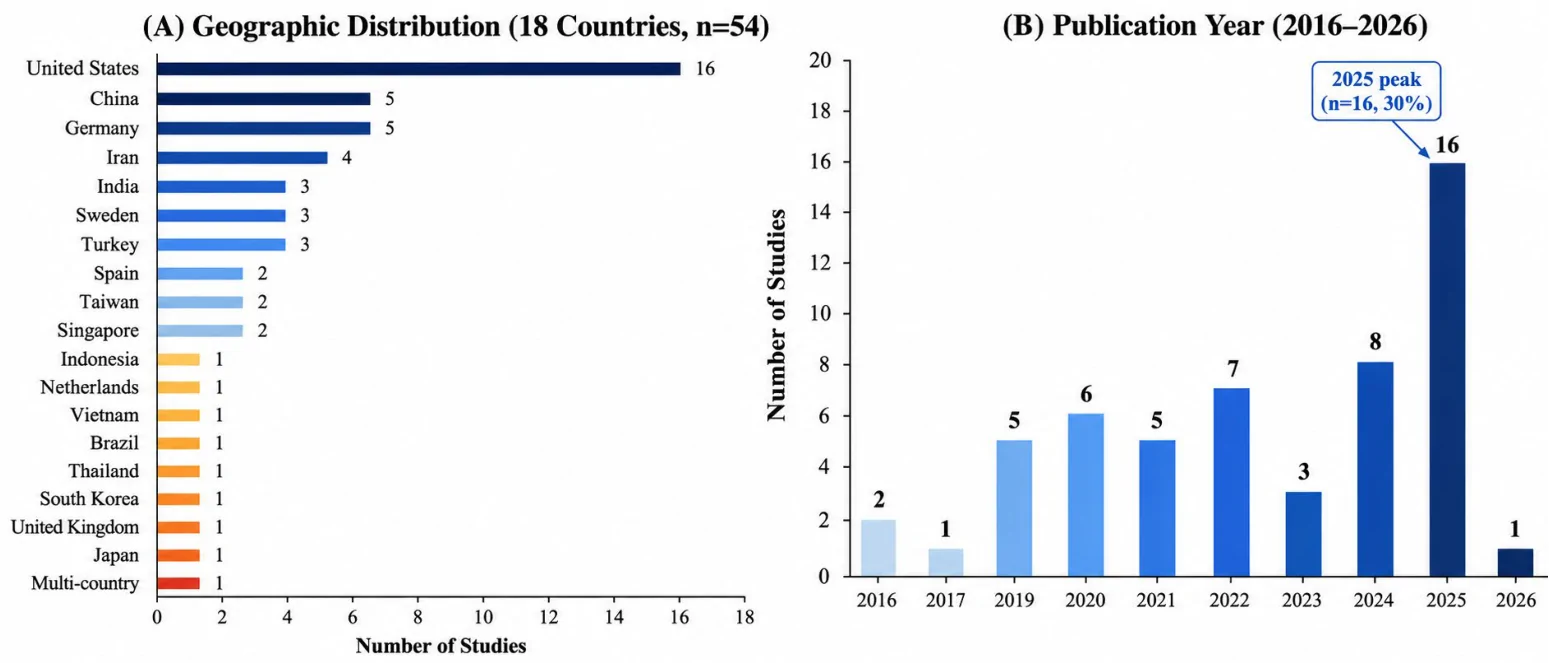
(**A**) Geographic distribution and (**B**) publication year trends.

The 54 studies spanned more than 18 countries (2016–2026). The most represented countries were the USA (*n* = 16, 30%), followed by China and Germany (*n* = 5, 9%), Iran (*n* = 4, 7%), and India, Sweden, and Turkey (*n* = 3, 6%), with further contributions from Taiwan, Spain, Singapore, Indonesia, Vietnam, South Korea, Japan, Brazil, the UK, and the Netherlands. Publication peaked in 2025 (*n* = 16, 30%), and 74% appeared after 2021, reflecting heightened attention to HCW mental health following the pandemic (Figure 2).

### 3.3. Study Designs, Intervention Types, and Program Components

**Figure 3:**
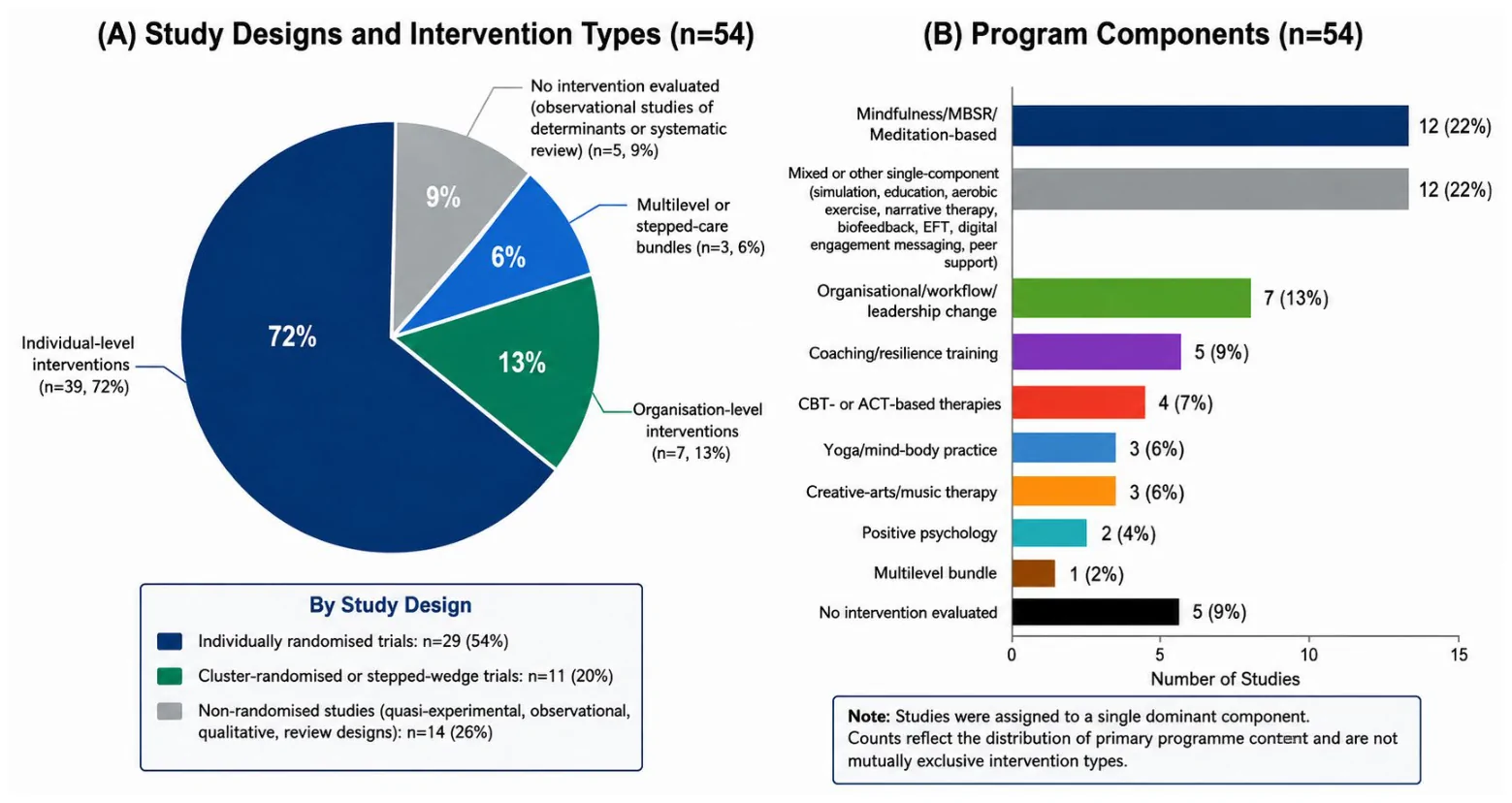
(**A**) Intervention taxonomy and (**B**) program component distribution.

By design, 29 studies (54%) were individually randomized trials, 11 (20%) were cluster-randomized or stepped-wedge trials, and 14 (26%) were non-randomized studies, including quasi-experimental intervention studies, observational studies, 1 qualitative study, 1 protocol paper, and 1 systematic review. Only eligible non-randomized intervention studies were assessed using ROBINS-I. By taxonomy, most studies evaluated individual-level interventions (*n* = 39, 72%), followed by organizational-level interventions (*n* = 7, 13%) and multilevel or stepped-care bundles combining both (*n* = 3, 6%). The remaining five studies (9%) did not evaluate an intervention, comprising four observational studies of determinants and one previously published systematic review (Figure 3A). Classifying studies by their dominant program component, the most common was mindfulness, MBSR, or meditation-based practice (*n* = 12, 22%), followed by mixed or other single-component programs (*n* = 12, 22%; comprising simulation and educational interventions, aerobic exercise, narrative therapy, biofeedback, emotional freedom technique, digital-engagement messaging, and peer support), organizational and workflow or leadership change (*n* = 7, 13%), coaching and resilience training (*n* = 5, 9%), CBT- or ACT-based therapies (*n* = 4, 7%), yoga and mind–body practice (*n* = 3, 6%), creative arts and music therapy (*n* = 3, 6%), positive psychology (*n* = 2, 4%), and one multilevel bundle (*n* = 1, 2%)—five studies evaluated no intervention (Figure 3B). Because many programs combined elements, each study was assigned to a single dominant component, and these counts should be read as the distribution of primary program content rather than as mutually exclusive intervention types.

### 3.4. Synthesis by Intervention Type and Program Component

**Figure 4:**
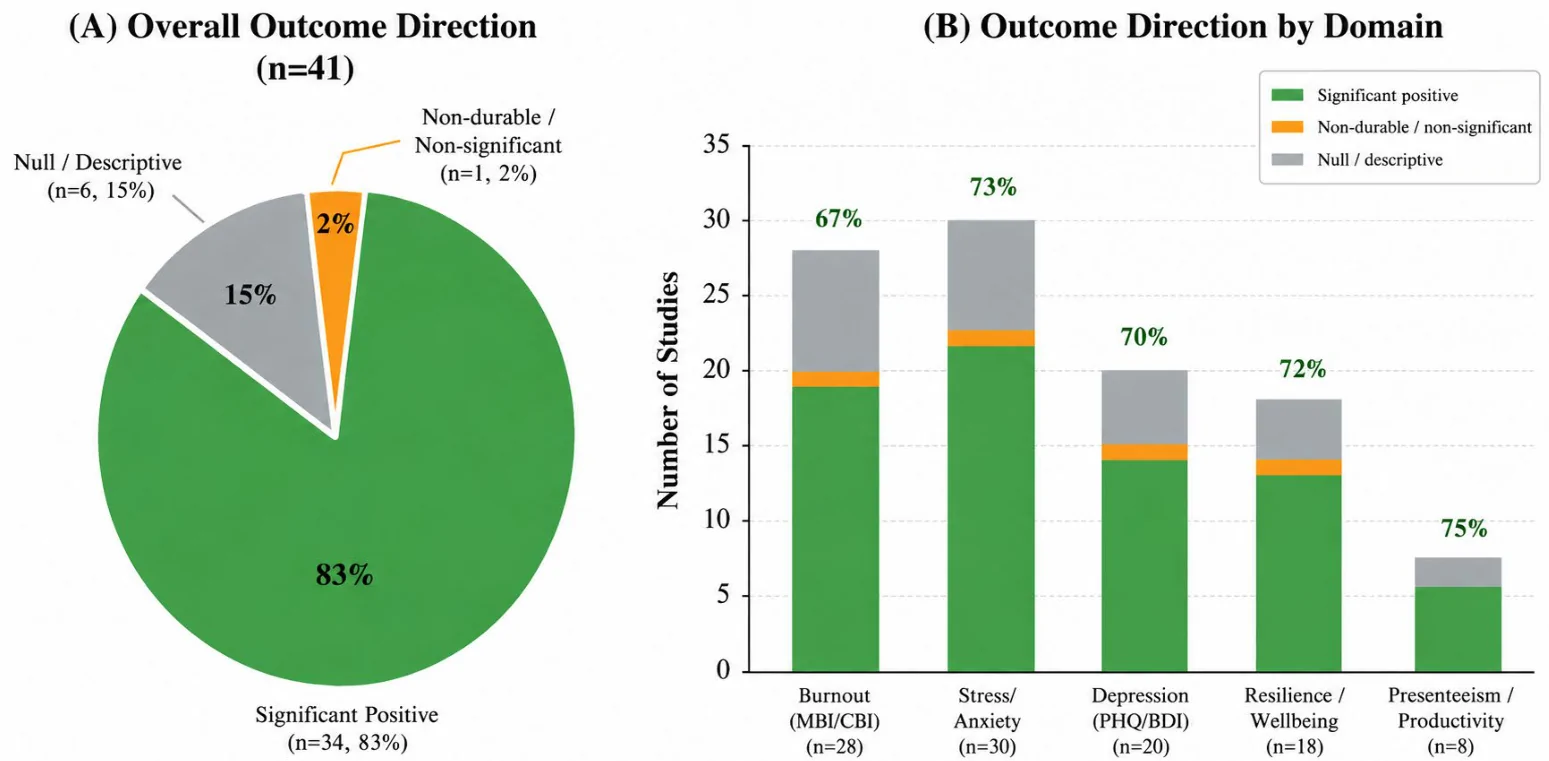
(**A**) Overall outcome direction and (**B**) by outcome domain.

Among the 41 effectiveness studies, 34 (83%) reported a statistically significant improvement in at least 1 primary or key mental health outcome, 6 (15%) reported a null result on their primary outcome, and 1 (2%) showed a non-durable effect. By domain, MBI/CBI instruments detected a significant positive finding in 67% of burnout studies (19 of 28), stress and anxiety in 73% (22 of 30), depression in 70% (14 of 20), resilience and wellbeing in 72% (13 of 18), and presenteeism and productivity in 75% (6 of 8). While statistically significant improvements were reported across multiple outcome domains, these findings should be interpreted cautiously because effect sizes, study quality, and the durability of benefits varied considerably across studies (Figure 4).

#### 3.4.1. Mindfulness- and MBSR-Based Interventions

Mindfulness-based interventions were the dominant category (*n* = 12, 22%), with the vast majority reporting significant positive outcomes across burnout, stress, anxiety, and wellbeing. Ameli et al. (2020; US RCT) found that the mindfulness-based self-care program significantly reduced perceived stress compared with the control group (*p* = 0.02), with sustained reductions at follow-up (Δ−6.14, *p* < 0.001) [15]. Wu et al. (2025; China RCT) found that online Mindfulness-Based Stress Reduction (MBSR) reduced anxiety (HADS-A −1.30, 95% CI −2.10 to −0.50, *p* = 0.002) and depression (HADS-D −2.05, 95% CI −2.89, −1.20, *p* < 0.001) [16], while Liu et al. (2025; China cluster RCT) demonstrated significant reductions in presenteeism (SPS-6: 16.63→11.95 at 12 weeks, *p* < 0.001) together with improvements in mindfulness (FFMQ: group effect, *p* = 0.002; time effect, *p* = 0.001) [17]. In a powered head-to-head trial, Arts-de Jong et al. (2025; Netherlands RCT) reported no superiority of facilitated MBSR versus self-guided digital MBI at six months (PHQ-SADS MD 0.23, *p* = 0.82) [18]. Both showed significant within-group improvements. This is a critical implementation finding: it suggests that resource-intensive facilitated MBSR offers no advantage over accessible, lower-cost digital mindfulness, with profound implications for scalability. Among mind–body and yoga interventions, Bhardwaj et al. (2023; India RCT) demonstrated that Sudarshan Kriya Yoga produced large burnout improvements (MBI all subscales, *d* = 0.87–0.90, *p* < 0.001), among the largest effect sizes in this review [19], and Celik and Kilinc (2022; Turkey RCT) found that laughter yoga significantly reduced stress (PSS: 32.47→21.39, *p* < 0.001) and burnout (MBI EE, *p* < 0.001) [20]. Yıldırım et al. (2022) showed reduced stress with mindfulness-based breathing and music therapy (*p* = 0.010) [21] (Table 1).

#### 3.4.2. Positive Psychology, Coaching, and Resilience Programs

Positive psychology and coaching approaches showed consistent effects, with seven studies (88%) reporting significant positive outcomes. Sexton and Adair (2024; US RCT) reported the strongest single effect estimate in the review, an emotional exhaustion reduction of −9.0 (95% CI: −13.1 to −4.9, *p* < 0.001) and emotional thriving +6.6 (*p* < 0.001) from brief continuing-education positive psychology modules [51]. The earlier Sexton et al. (2022) RCT confirmed significant depressive symptom reduction (−7.5, *p* < 0.001) and work–life integration improvements at one week [50]. These Two Good Things/gratitude modules are exceptionally brief (1–3 weeks) yet produce clinically meaningful effect sizes. Fainstad et al. (2022; US pilot RCT) found that group coaching reduced emotional exhaustion (EE −4.33, CI −7.64 to −1.01, *p* = 0.01) and depersonalization in physicians [26]. Bock et al. (2025; Germany RCT) found that digital resilience “learning nuggets” significantly increased resilience (RS-Scale, d = 0.60, *p* < 0.001), with a large stress reduction [60]. Super et al. (2024; UK RCT) found that self-compassion training produced significant improvements in wellbeing (ηp^2^ = 0.135, *p* < 0.001) and perceived stress reduction [67] (Table 1).

#### 3.4.3. CBT, ACT, and Structured Psychological Therapies

CBT- and ACT-based programs were effective where used, though they form a smaller part of the evidence base. Zhang et al. (2024; China RCT) produced the largest effect size for distress (DASS-21 *β* = −3.85 to −7.50, *d* up to 1.40, *p* < 0.001) [32]. Mediavilla et al. (2023; Spain multicenter RCT) demonstrated that a stepped-care psychological intervention beginning with a WHO digital guide (“Doing What Matters in Times of Stress”) produced significant reductions in the combined PHQ-ADS (difference 4.4, standardized effect size 0.8, *p* < 0.001), demonstrating that low-intensity stepped-care is effective even in complex multicenter HCW populations [64]. Imamura et al. (2021; Vietnam three-arm RCT) found that iCBT reduced depression at three months (*p* = 0.048) but not at seven months, indicating decay of digital CBT effects without boosters [44]. Similarly, López-del-Hoyo et al. (2025; Spain) found that a third-wave psychotherapy intervention significantly reduced perceived stress (*β* = −1.08; 95% CI −2.08 to −0.07; *p* < 0.05), supporting the effectiveness of structured psychological therapies in reducing distress among healthcare workers [30] (Table 1).

#### 3.4.4. Organizational Interventions: A Thinner and Less Consistent Evidence Base

Organizational interventions were more heterogeneous and far less consistently evaluated than individual programs, and this contrast is a central finding of the review. Linzer et al. (2019; US prospective cohort) reported that higher organizational trust was associated with lower burnout across multiple dimensions (all *p* < 0.05) [35]. These findings suggest that organizational factors, such as trust, may play an important role in workforce wellbeing; however, because the evidence is derived from observational research, causal inferences cannot be drawn. Furthermore, non-intervention observational studies were retained only as contextual evidence and were not included in the ROBINS-I assessment, which was restricted to eligible non-randomized intervention studies. Prasad et al. (2019; USA cluster RCT) similarly reported that reducing workplace time pressure was associated with lower stress (effect size = 0.417; *p* = 0.015), burnout (effect size = 0.384; *p* = 0.017), and intention to leave (effect size = 0.345; *p* = 0.046) [34]. Belkic (2025; India IPD analysis) demonstrated that increasing occupational stress index scores significantly increased personal burnout (OR = 1.11; 95% CI 1.03–1.18; *p* = 0.003), work-related burnout (OR = 1.17; 95% CI 1.08–1.26; *p* = 0.0001), and patient-related burnout (OR = 1.07; 95% CI 1.01–1.15; *p* = 0.03) [55]. Brannstrom et al. (2025; Sweden cluster RCT) found that ethics communication groups improved the ethical climate (*p* = 0.036) without significantly reducing moral distress [65]. Degen et al. (2021; Germany cluster RCT) found that participatory leadership workshops improved job satisfaction differentially by employment status [41]. Critically, the two most rigorous bundle RCTs, Mulfinger et al. (2025; Germany) [57] and Bjorkelund et al. (2024; Sweden) [22], found no significant differences versus comparators on primary burnout outcomes. This may reflect the challenges of simultaneously implementing organizational changes while delivering individual programs, or that individual-level improvements mask residual organizational stressors (Table 1).

#### 3.4.5. Delivery Mode

**Figure 5:**
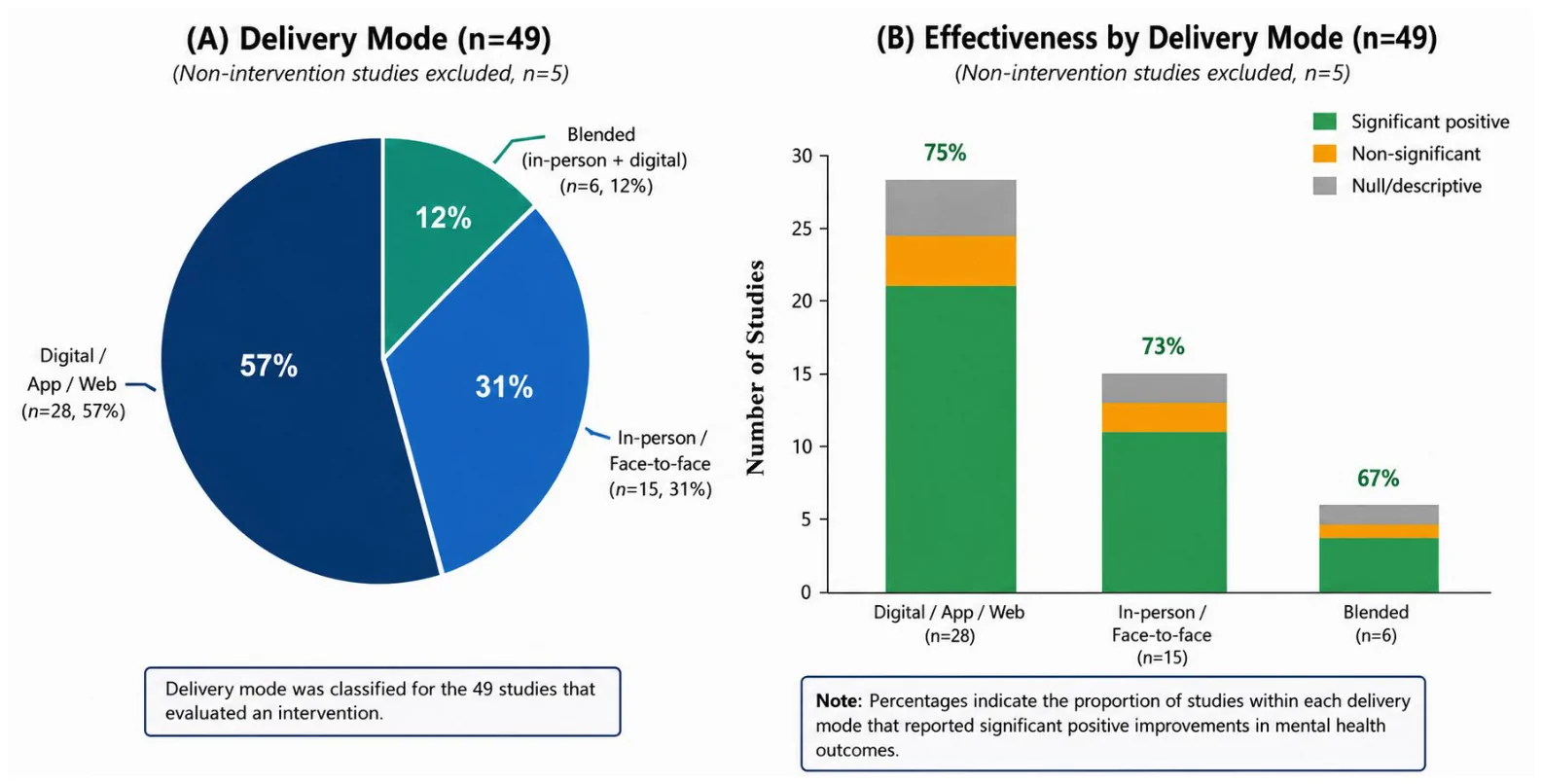
(**A**) Delivery mode distribution and (**B**) effectiveness by mode.

Delivery mode was classified for the 49 studies that evaluated an intervention, and 5 non-intervention studies were excluded. Digital delivery (app- or web-based) was the most common mode (*n* = 28, 57%), followed by face-to-face delivery (*n* = 15, 31%) and blended delivery combining digital and in-person components (*n* = 6, 12%). This distribution reflects the increasing adoption of remotely delivered interventions between 2016 and 2026, particularly during and after the COVID-19 pandemic. Comparable proportions of digital and face-to-face interventions reported significant improvements in mental health outcomes, suggesting that digital delivery offers similar effectiveness while improving accessibility and scalability for healthcare workers (Figure 5).

### 3.5. Outcome of the Risk-of-Bias Assessment

**Figure 6:**
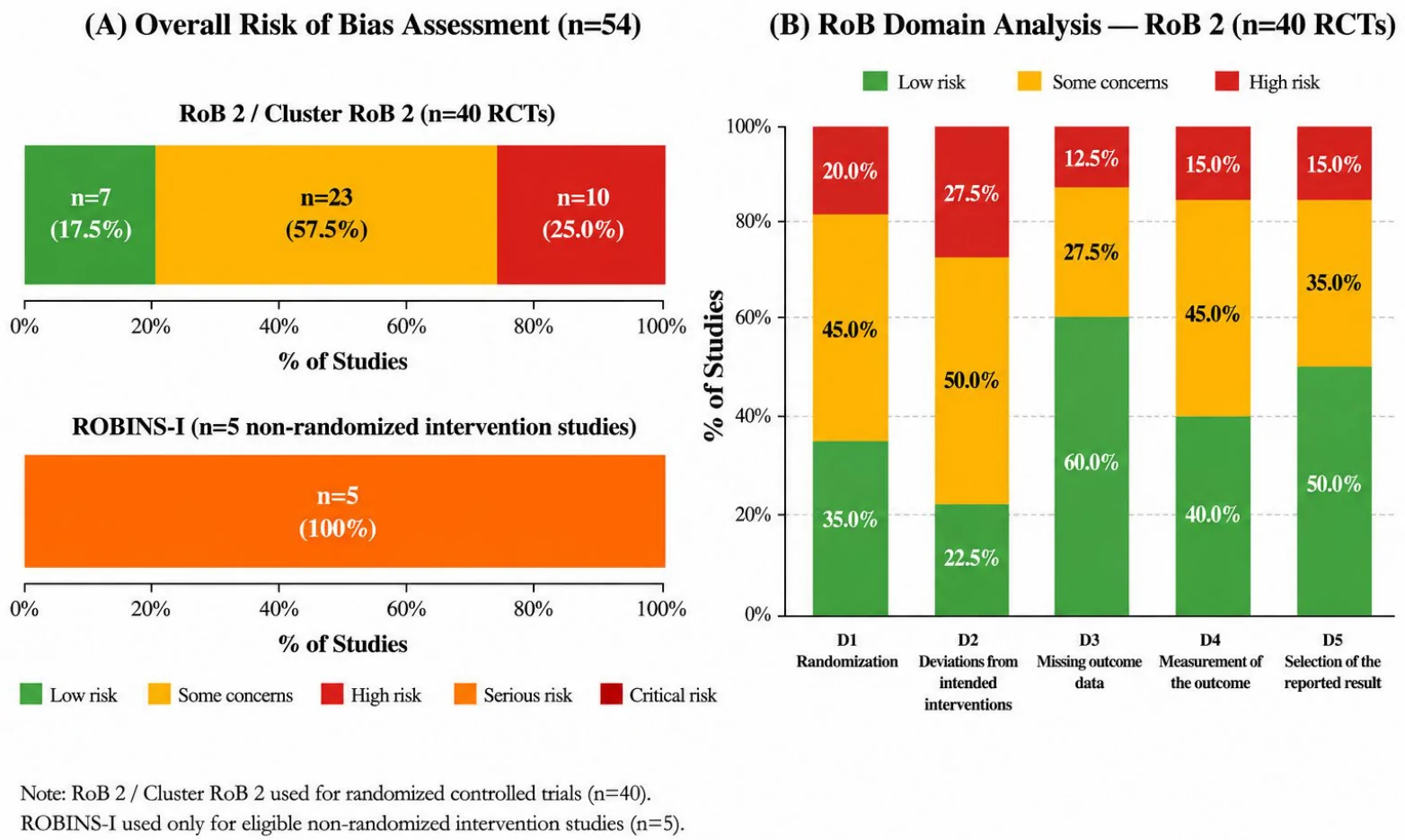
Risk-of-bias assessment summary: (**A**) overall and (**B**) domain level.

Of the 54 included studies, 40 randomized controlled trials (74.1%) were assessed using RoB 2/Cluster RoB 2, of which 7 (17.5%) were rated as low risk, 23 (57.5%) as having some concerns, and 10 (25.0%) as high risk. ROBINS-I was applied only to the five eligible non-randomized intervention studies. All five studies were judged to have serious risk of bias, with none rated as low, moderate, or critical risk. Non-randomized studies that did not evaluate intervention effects, together with qualitative studies, protocol papers, and systematic reviews, were retained as contextual evidence but were not included in the ROBINS-I assessment. Domain-level analysis of the randomized trials showed that missing outcome data (D3) had the highest proportion of low-risk judgments (60.0%), whereas deviations from intended interventions (D2) had the greatest proportion of studies with some concerns (50.0%) and the highest proportion of high-risk ratings (27.5%). Measurement of the outcome (D4) also showed substantial concerns, with 45.0% of studies rated as having some concerns and 15.0% as high risk, while randomization (D1) and selection of the reported result (D5) demonstrated low-risk ratings of 35.0% and 50.0%, respectively (Figure 6).

### 3.6. Certainty of Evidence (GRADE-Informed)

The certainty of evidence ranged from very low to moderate. Mindfulness/MBSR, positive psychology, iCBT/ACT, and digital versus in-person MBSR showed moderate-certainty evidence with generally consistent positive outcomes. Resilience and self-compassion interventions demonstrated low-to-moderate certainty evidence for improving wellbeing. Evidence for combined individual and organizational interventions was low certainty due to inconsistent findings, while organizational workflow and trust-based interventions had very-low-certainty evidence, being supported mainly by observational studies (Table 2).

## 4. Discussion

This synthesis of 54 studies provides the most comprehensive comparison to date of individual, organizational, and multilevel approaches to HCW mental health across more than 18 countries. Among the 41 studies contributing effectiveness evidence, 34 (83%) reported at least 1 statistically significant positive outcome. However, this proportion should be interpreted cautiously because studies reporting favorable results are generally more likely to be published than those reporting null or negative findings, potentially overestimating intervention effectiveness. As a formal assessment of publication bias was not feasible without a meta-analysis, the true balance of intervention effects may be less favorable than the published evidence suggests. Moreover, statistical significance does not necessarily indicate overall intervention effectiveness, as effect sizes, durability of benefits, primary versus secondary outcomes, and methodological quality varied considerably across studies. Several studies also had small sample sizes, short follow-up periods, or a high risk of bias. Collectively, these findings suggest potential benefits across multiple outcome domains but do not establish consistent effectiveness across all intervention types. More importantly, from a sociology of work perspective, the asymmetry of the evidence base highlights where responsibility for promoting healthy work environments continues to be placed.

### 4.1. Individual Interventions Work, but the Question Has Moved On

The retrieved PubMed-indexed evidence generally suggests that mindfulness, CBT, and positive psychology interventions may improve burnout, stress, and psychological distress outcomes among healthcare workers, although the certainty and durability of these effects vary across studies. The live questions are now more specific. First, do gains persist? Most studies measured one to six months, and decay is documented for iCBT without boosters [44]. Second, which components drive the effect? Sexton’s finding that a one-week module produces emotional exhaustion reductions comparable to eight-week MBSR suggests program intensity is not the primary determinant [50,51]. Third, is digital equivalent to in-person? The Arts-de Jong et al. (2025) [18] equivalence trial indicates that it is. These are valuable findings, but they all concern how to optimize interventions that leave working conditions untouched.

### 4.2. The Organizational Intervention Gap

The most consequential finding is the contrast between the robustness of individual intervention evidence and the poverty of organizational evidence. Only 10 organizational studies were identified, of which 6 were RCTs, and the most rigorous studies conducted by Mulfinger et al. (2025) [57] and Bjorkelund et al. (2024) [22] reported null results. The observational evidence presented by Linzer et al. (2019) [35] and Belkic (2025) [55] persuasively identifies workload, trust, and structural stressors as potent burnout determinants, yet cannot support causal recommendations without randomized confirmation. This gap is not merely academic. If workload, understaffing, and moral injury are important structural contributors to burnout, individual interventions that leave these structures unchanged may reduce symptoms without fully addressing underlying organizational contributors and may contribute to a framing in which the worker, rather than the organization of work, is viewed as the primary site of intervention. The field urgently needs adequately powered RCTs of workload reduction, schedule flexibility, and participatory leadership with burnout as the primary outcome and sufficient follow-up to detect it.

### 4.3. Digital Delivery as a Scalability Solution, Not a Substitute for Structural Reform

Available evidence suggests that digital interventions may provide outcomes comparable to face-to-face approaches while improving accessibility and scalability; however, the evidence base remains limited. Digital platforms overcome the shift-work, time-pressure, geographic, and stigma-related barriers faced by healthcare workers, as demonstrated by Coelhoso et al. (2019) [52], Wu et al. (2025) [16], Liu et al. (2025) [17], Agarwal et al. (2024) [49], and Bock et al. (2025) [60], without sacrificing effectiveness. Health systems should treat digital mental health provision as preventive infrastructure rather than a second-best option. At the same time, scalable individual provision should not be mistaken for, or substituted for, reform of the conditions that generate distress in the first place.

### 4.4. Implementation Evidence: What Enables Success

Qualitative and mixed-methods studies identify enablers, protected time [36], leadership buy-in and visible participation [37,53], peer-champion models, and opt-out enrolment, and barriers, time constraints, shift incompatibility, stigma, and the perceived irrelevance of generic wellness content to clinical work. Content should be contextualized to HCW-specific stressors (moral injury, patient death, and peer loss). On this basis we suggest deploying digital mindfulness and positive psychology programs as standard infrastructure, favoring brief, high-impact formats (1–4 sessions), mandating organizational stressor auditing with validated instruments (OSI and COPSOQ), funding powered RCTs of working condition reforms, and including retention outcomes (turnover intention, attrition, absenteeism, and presenteeism) as co-primary endpoints in future trials.

### 4.5. Limitations

The findings should be interpreted in light of several limitations. First, the literature search was restricted to PubMed/MEDLINE, and relevant studies indexed exclusively in other databases may have been missed, particularly organizational intervention studies. Second, substantial heterogeneity in interventions, outcome measures, and follow-up periods precluded meta-analysis. Third, most studies had short follow-up durations (1–6 months), limiting assessment of long-term effects. Fourth, the predominance of individual-level interventions may reflect their greater feasibility for randomized evaluation, potentially contributing to an imbalance in the evidence base. Many studies also enrolled self-selected participants, limiting generalizability. In addition, the review was not prospectively registered, reducing protocol transparency. Finally, the certainty of the evidence was limited by methodological quality, with relatively few randomized trials at low risk of bias and all five eligible non-randomized intervention studies assessed using ROBINS-I judged to have a serious risk of bias.

## 5. Conclusions

Within the PubMed/MEDLINE-indexed literature included in this review, individual-level interventions, such as mindfulness-based programs, positive psychology approaches, coaching, and digital psychological interventions, were frequently associated with improvements in burnout, stress, and psychological distress outcomes. However, these findings should be interpreted cautiously because of methodological heterogeneity, substantial risk of bias in many included studies, and the restriction of the search strategy to a single database. Evidence regarding organizational interventions remains limited and inconsistent, highlighting the need for additional high-quality studies before firm conclusions can be drawn regarding their effectiveness.

Based on the evidence identified in this review, future research should prioritize rigorous evaluations of organizational interventions, including workload, staffing, and leadership reforms, using adequately powered randomized controlled trials with longer follow-up periods. Future studies should also incorporate validated measures of burnout, retention, and organizational outcomes to strengthen the certainty and applicability of the evidence. Although digital and individual-level interventions appear promising within the included studies, additional high-quality comparative research is needed before recommending their widespread implementation as standard practice.

## Figures and Tables

**Table 1:** Summary of evidence from included studies.

No.	Author (Year)	Country	Design	Intervention	Burnout Scale	Key Findings	RoB
1	Bhardwaj et al. (2023) [19]	India	RCT	Individual: SKY Yoga/mind–body	MBI-HSS	Burnout MBI all subscales: *p* < 0.001, *d* = 0.87–0.90 (large); ProQOL improved	Some Concerns
2	Björkelunt et al. (2024) [22]	Sweden	Cluster RCT	Org: collaborative care + workplace dialogue	KEDS	NS in sick leave days; gross sick leave 160 vs. 99 (*p* = n.s.)	Some Concerns
3	Andani et al. (2025) [23]	Indonesia	Cluster RCT	Bundle: music therapy + org	MBI-HSS	Burnout ↓ weeks 2, 4 (*p* = 0.001–0.022); HRV improved	Some Concerns
4	Evans et al. (2025) [24]	United States	RCT	Individual: simulation training	HRV (physiologic stress proxy)	HRV post-intervention: mean difference 11.55 ms (*p* = 0.007) lower stress	Low
5	Chang et al. (2022) [25]	Taiwan	Cluster RCT	Org: workplace violence training	OCSE, goal commitment	Goal commitment Δ +4.1 vs. +0.08 (*p* < 0.001); OCSE Δ +7.0	Some Concerns
6	Fainstad et al. (2022) [26]	United States	Pilot RCT	Individual: professional coaching	MBI	EE −4.33 (CI −7.64–−1.01; *p* = 0.01); DP significantly improved	High
7	Ameli et al. (2020) [15]	United States	RCT	Individual: MBSC (mindfulness)	MBI 2-item	Stress PSS: 17.29 vs. 18.54 (*p* = 0.02); Δ−6.14 at f/u (*p* < 0.001)	Some Concerns
8	Çelik and Kılınç (2022) [20]	Turkey	RCT	Individual: laughter yoga	MBI	PSS: 32.47 → 21.39 (*p* < 0.001); MBI EE ↓ (*p* < 0.001)	Some Concerns
9	Saeidavi et al. (2025) [27]	Iran	Quasi-experimental	Individual: occupational stress education	Occupational stress scale	Pressure ↓ (*p* = 0.012, *d* = −0.305); occupational stress ↓	Some Concerns
10	Dincer et al. (2020) [28]	Turkey	RCT	Individual: EFT (tapping)	Burnout scale	Stress SUD ↓ (*p* < 0.001); anxiety STAI ↓ (*p* < 0.001)	Some Concerns
11	Yıldırım et al. (2022) [21]	Turkey	RCT	Individual: mindfulness breathing	None direct (stress only)	Stress STAI ↓ (*p* = 0.010); psychological wellbeing ↑ (*p* < 0.05)	Some Concerns
12	Wu et al. (2025) [16]	China	RCT	Individual: online MBSR	None direct (anxiety/depression)	Anxiety HADS: −1.30 (CI −2.10–−0.50, *p* =0.002); depression ↓	Some Concerns
13	Antico and Brewer et al. (2025) [29]	United States	Single-arm pilots	Individual: digital mindfulness training	MBI (cynicism/EE)	33% cynicism reduction (*p* = 0.04, *r* = 0.41); EE ↓	Critical
14	López-del-Hoyo et al. (2025) [30]	Spain	Cluster RCT	Individual: self-guided digital (3rd wave)	None direct (stress)	Perceived stress *β* = −1.08 (*p* = 0.03); per-protocol *β* = −1.84 (*p* = 0.003)	Some Concerns
15	Joshi et al. (2022) [31]	United States	RCT	Individual: Transcendental Meditation	MBI	GSI NS; MBI EE significant (*p* < 0.05)	Some Concerns
16	Zhang et al. (2024) [32]	China	RCT	Individual: iACT (internet-ACT)	MBI-GS	Distress DASS-21 *β* = −3.85 to −7.50 (*p* < 0.001; *d* up to 1.40)	High
17	Abbasalizadeh et al. (2024) [33]	Iran	RCT	Individual: resilience skills training	None direct (stress/anxiety)	Stress ↓ (*p* = 0.001); anxiety STAI ↓ (*p* = 0.001)	Low
18	Prasad et al. (2019) [34]	United States	Prospective evaluation (intervention)	Org: workflow redesign	Burnout/satisfaction scales	67-time pressure; gender differences (women higher, *p* < 0.05)	Serious
19	Linzer et al. (2019) [35]	United States	Prospective cohort	Org: trust-based workflow redesign	Burnout scale	High trust → lower burnout; all organizational factors *p* < 0.05	Not applicable
20	Hersch et al. (2016) [36]	United States	RCT	Individual: web-based stress management	Nursing Stress Scale	NSS full: *t* = −2.95, *p* = 0.003; multiple subscales significant	High
21	Rossetti et al. (2023) [37]	United States	Observational/RCT baseline	Bundle: environmental music therapy	Staff stress perception	APACHE II ↓ (*p* < 0.001); pain ↓ (*p* = 0.022); sleep improved	Serious
22	Gao et al. (2024) [38]	China	RCT	Individual: digital music + self-help	DASS-21	DASS total NS (*p* = 0.147); depression secondary NS (*p* = 0.072)	Low
23	Mandal et al. (2021) [39]	India	RCT	Individual: structured yoga	ProQOL, PSS	PSS: 15.4 vs. 20.7 mean (*p* < 0.001); ProQOL compassion satisfaction ↑	High
24	Arts-de Jong et al. (2025) [18]	Netherlands	RCT	Individual: MBSR (adjusted)	PHQ-SADS composite	MBSR vs. MBI: NS (MD 0.23, *p* = 0.82); within-group both ↑	Low
25	Pieper et al. (2025) [40]	Germany	Multicenter RCT	Individual: creative arts therapy	CBI, job satisfaction	Job satisfaction NS (*p* > 0.05); CBI subscales NS at 3 months	High
26	Degen et al. (2021) [41]	Germany	Cluster RCT	Org: leadership workshops + participatory	COPSOQ	Job satisfaction: practice owners vs. assistants (*p* < 0.01); chronic stress ↓	Low
27	Teo et al. (2021) [42]	Singapore	Prospective cohort (observational)	Observational: no intervention	Single-item burnout, stress	Stress +1%/month (*p* < 0.05); burnout +1.2%/month (*p* < 0.01)	Not applicable
28	Mohebbi et al. (2019) [43]	Iran	Controlled clinical trial	Individual: aerobic exercise	Occupational stress (HSE)	HSE ↑ post: 119.7 vs. 86.2 (*p* < 0.001) → improved stress management	Some Concerns
29	Imamura et al. (2021) [44]	Vietnam	Three-arm RCT	Individual: digital iCBT (2 formats)	Depression/anxiety	Program B: depression ↓ at 3 months (*p* = 0.048, *d* = −0.18); NS at 7 months	Some Concerns
30	Xue et al. (2025) [45]	China	Cluster RCT	Individual: narrative therapy	PCL-5 (PTSD)	PTSD total ↓ (*p* = 0.025); criteria B, D, E significant	Some Concerns
31	Ho et al. (2019) [46]	Singapore	Trial RCT	Individual: MCAT (mindfulness + art)	MBI-GS	MCAT protocol paper; outcomes hypothesized (trial ongoing at publication)	Not applicable
32	Pihlgren et al. (2024) [47]	Sweden	Qualitative IPA	Individual: ICTI memory intervention	Intrusive memories	Intrusive memories vivid/distressing; ICTI perceived acceptable + helpful	Not applicable
33	Bernburg et al. (2016) [48]	Germany	Cross-sectional survey	Observational: COPSOQ survey	COPSOQ burnout subscale	17% high distress; mean burnout 0.48; work demand key driver (*p* < 0.05)	Not applicable
34	Agarwal et al. (2024) [49]	United States	RCT (9-month)	Individual: proactive digital push	Wellbeing Index (WBI)	Depression + anxiety ↓ significantly at 9 months (*p* < 0.01)	Some Concerns
35	Sexton et al. (2022) [50]	United States	RCT	Individual: positive psychology modules	MBI-derived 5-item	Depressive symptoms −7.5 (*p* < 0.001); EE improved at 1 week	High
36	Sexton and Adair et al. (2024) [51]	United States	RCT	Individual: positive psychology (CME)	MBI-derived 5-item	EE −9.0 (CI −13.1–−4.9, *p* < 0.001); emotional thriving +6.6 (*p* < 0.001)	Some Concerns
37	Coelhoso et al. (2019) [52]	Brazil	RCT	Individual: digital self-help app	PSS-10, WHO-5	PSS-10 ↓ −6.15 (clinically significant); reaches symptom-free threshold	High
38	Timmins et al. (2025) [53]	United States	Mixed-methods nested	Individual: peer support (SFA)	Acceptability survey	48.2% helpful; 51.2% recommend; 59.4% comfortable supporting peers	Serious
39	Srikasem et al. (2025) [54]	Thailand	Quasi-experimental	Individual: multimodal education (ER-VIPE)	CBI	DSSQ-engagement ↑ Group B (*p* = 0.001); worry ↓	Serious
40	Belkić (2025) [55]	India (AIIMS)	IPD analysis	Org: work conditions (OSI)	CBI	OSI ↑ → burnout OR 1.11–1.17 (*p* = 0.003); work overload dominant driver	Not applicable
41	Janicki et al. (2020) [56]	United States	Prospective observational	Observational: physiologic monitoring	HR, HRV, single-item burnout	HR ↑ 70 → 78 bpm during shifts (*p* < 0.001); HRV ↓ during shifts	Not applicable
42	Mulfinger et al. (2025) [57]	Germany	Cluster RCT	Bundle: individual + org (multilevel)	IRR scale	IRR NS (ANCOVA IG vs. WCG: −0.51, *p* > 0.05); WHO-5 both declined	High
43	Benzo et al. (2017) [58]	United States	Cross-sectional	Observational: self-compassion factors	Stress/wellbeing (no MBI)	Self-compassion explains 39% variance in happiness; resilience mediates	Not applicable
44	Pollock et al. (2020) [59]	Multi-country	Mixed methods review	Individual: psychological first aid	ProQOL burnout	Very low certainty; PFA reduced burnout	Not applicable
45	Bock et al. (2025) [60]	Germany	RCT (waitlist)	Individual: digital resilience “nuggets”	RS-Scale, PSQ	Resilience ↑ (*p* < 0.001, *d* = 0.6); Stress ↓ (*p* <0.001, large effect)	Low
46	Duva et al. (2022) [61]	United States	RCT	Individual: CRM (somatic resilience)	UWES, WHO-5	WHO-5 ↑ (*p* < 0.0087, *d* = 0.66); WEMWBS ↑ (*p* < 0.0004); teamwork ↑	High
47	Hsieh et al. (2020) [62]	Taiwan	Quasi-experimental cluster	Individual: biofeedback + SDBT	Resilience, depression	Depression ↓ (*p* < 0.001); resilience ↑ (*p* < 0.001)	Some Concerns
48	Liu et al. (2025) [17]	China	Cluster RCT	Individual: MBSR (8-week)	SPS-6 (presenteeism)	Presenteeism ↓ 16.63 → 11.95 at 12 weeks (*p* < 0.001)	Some Concerns
49	Lee et al. (2020) [63]	South Korea	Non-randomized controlled	Individual: online mind–body training	SRI, CDRS (resilience)	Stress ↓ at 8 + 12 weeks (*p* < 0.05); resilience ↑ sustained	Serious
50	Mediavilla et al. (2023) [64]	Spain	Multicenter RCT	Individual: stepped-care psychological	PHQ-ADS composite	PHQ-ADS diff 4.4 (CI 2.1–6.7, SES = 0.8, *p* < 0.001); both PHQ-9 + GAD-7	Some Concerns
51	Brännström et al. (2025) [65]	Sweden	Cluster RCT	Org: ethics communication groups (ECG)	MMD-HP, moral distress	Ethical climate ↑ at 3 + 6 months (*p* = 0.036); moral distress NS	Some Concerns
52	Azizi et al. (2026) [66]	Iran	RCT	Individual: resilience enhancement program	CD-RISC-25	Resilience ↑ post-test + 6-month f/u (*p* = 0.028–0.029)	Some Concerns
53	Super et al. (2024) [67]	United Kingdom	RCT	Individual: self-compassion training	Self-compassion, wellbeing	Self-compassion *F* = 32.72 (*p* < 0.001); wellbeing *F* = 17.46 (*p* < 0.001)	High
54	Imai et al. (2021) [68]	Japan	RCT	Individual: presenteeism intervention	SPS-6 (presenteeism)	Absolute presenteeism ↑ (*F* = 12.87, *p* = 0.001); pain ↓ significantly	Low

MBSR = Mindfulness-Based Stress Reduction; MBI = Maslach Burnout Inventory; MBI-HSS = Human Services Survey; MBI-GS = General Survey; CBI = Copenhagen Burnout Inventory; ProQOL = Professional Quality of Life; PSS = Perceived Stress Scale; PHQ = Patient Health Questionnaire; GAD = Generalized Anxiety Disorder scale; DASS = Depression Anxiety Stress Scales; PSQ = Perceived Stress Questionnaire; RS-Scale = Resilience Scale; UWES = Utrecht Work Engagement Scale; WEMWBS = Warwick-Edinburgh Mental Wellbeing Scale; SPS = Stanford Presenteeism Scale; CRM = Community Resiliency Model; iACT = internet-ACT; MCAT = Mindfulness Compassion Art Therapy; ICTI = Intrusive Cognitive Trauma Intervention; OCSE = Occupational Coping Self-Efficacy; IRR = Irritation Scale; ECG = Ethics Communication Groups; HRV = Heart Rate Variability; SFA = Stress First Aid; EFT = Emotional Freedom Techniques; SKY = Sudarshan Kriya Yoga; ↓-decreased, ↑-increased. RoB column: RoB 2 was applied to randomized trials, Cluster RoB 2 to cluster-randomized/stepped-wedge trials, and ROBINS-I to eligible non-randomized intervention studies only. Studies not evaluating intervention effects (including protocol, qualitative, review, and non-intervention observational studies) are marked as “not applicable”.

**Table 2:** GRADE-informed certainty of evidence by intervention and outcome.

Intervention/Outcome	Certainty	Rationale	Direction
Mindfulness/MBSR → burnout reduction	Moderate	Multiple RCTs demonstrated consistent reductions in burnout and stress, although heterogeneity in intervention format and outcome measures and reliance on self-reported outcomes resulted in downgrading [15,16,17,18,19,20].	Consistently positive
Positive psychology → emotional exhaustion	Moderate	Two sequential randomized trials reported consistent improvements in emotional exhaustion and wellbeing, but certainty was downgraded for single-country evidence and short follow-up [50,51].	Consistently positive (US evidence)
iCBT/ACT → psychological distress	Moderate	Randomized trials of internet-based ACT and CBT consistently reduced psychological distress, although replication across settings remains limited [30,32,44,64].	Positive (moderate)
Digital vs. in-person MBSR	Moderate	One adequately powered equivalence trial supported similar effectiveness of digital and face-to-face delivery, supported by additional digital intervention studies [16,17,18,52].	Equivalent (neither superior)
Organizational workflow/trust → burnout	Very low	Evidence is derived primarily from observational studies with substantial risk of confounding and limited randomized evidence [34,35,55].	Positive (observational only)
Bundle (individual + organizational) → burnout	Low	Two cluster RCTs found no significant benefit, whereas one bundle intervention reported positive findings, resulting in inconsistent evidence [22,23,57].	Inconclusive/null in RCTs
Resilience/self-compassion → wellbeing	Low–moderate	Randomized trials consistently reported improvements in resilience and wellbeing, although intervention heterogeneity limited certainty [33,60,66,67].	Mostly positive

## Data Availability

All extracted data are available upon reasonable request.

## Supplementary Materials

The following supporting information can be downloaded at: https://www.mdpi.com/article/10.3390/ijerph23081002/s1. Table S1: Search Strategy; Table S2: Datas; Table S3: PRISMA Checklist, Table S4: Risk of Bias.

## References

[1] Office of the Surgeon General (OSG) (2022). Addressing Health Worker Burnout: The U.S. Surgeon General’s Advisory on Building a Thriving Health Workforce.

[2] De Hert S. (2020). Burnout in Healthcare Workers: Prevalence, Impact and Preventative Strategies. Local Reg. Anesth..

[3] Lai J., Ma S., Wang Y., Cai Z., Hu J., Wei N., Wu J., Du H., Chen T., Li R. (2020). Factors Associated with Mental Health Outcomes Among Health Care Workers Exposed to Coronavirus Disease 2019. JAMA Netw. Open.

[4] Aljabri D., Alshatti F., Alumran A., Al-Rayes S., Alsalman D., Althumairi A., Al-kahtani N., Aljabri M., Alsuhaibani S., Alanzi T. (2022). Sociodemographic and Occupational Factors Associated with Burnout: A Study Among Frontline Healthcare Workers During the COVID-19 Pandemic. Front. Public Health.

[5] Hall L.H., Johnson J., Watt I., Tsipa A., O’Connor D.B. (2016). Healthcare Staff Wellbeing, Burnout, and Patient Safety: A Systematic Review. PLoS ONE.

[6] Shanafelt T.D., Noseworthy J.H. (2017). Executive Leadership and Physician Well-Being: Nine Organizational Strategies to Promote Engagement and Reduce Burnout. Mayo Clin. Proc..

[7] West C.P., Dyrbye L.N., Erwin P.J., Shanafelt T.D. (2016). Interventions to Prevent and Reduce Physician Burnout: A Systematic Review and Meta-Analysis. Lancet.

[8] Guidelines on Mental Health at Work. https://www.who.int/publications/i/item/9789240053052.

[9] Gabriel K.P., Aguinis H. (2022). How to Prevent and Combat Employee Burnout and Create Healthier Workplaces during Crises and Beyond. Bus. Horiz..

[10] Lomas T., Medina J.C., Ivtzan I., Rupprecht S., Eiroa-Orosa F.J. (2018). A Systematic Review of the Impact of Mindfulness on the Well-Being of Healthcare Professionals. J. Clin. Psychol..

[11] Guo Y.-F., Luo Y.-H., Lam L., Cross W., Plummer V., Zhang J.-P. (2018). Burnout and Its Association with Resilience in Nurses: A Cross-Sectional Study. J. Clin. Nurs..

[12] Montgomery A., Todorova I., Baban A., Panagopoulou E. (2013). Improving Quality and Safety in the Hospital: The Link between Organizational Culture, Burnout, and Quality of Care. Br. J. Health Psychol..

[13] Page M.J., McKenzie J.E., Bossuyt P.M., Boutron I., Hoffmann T.C., Mulrow C.D., Shamseer L., Tetzlaff J.M., Akl E.A., Brennan S.E. (2021). The PRISMA 2020 Statement: An Updated Guideline for Reporting Systematic Reviews. BMJ.

[14] Campbell M., McKenzie J.E., Sowden A., Katikireddi S.V., Brennan S.E., Ellis S., Hartmann-Boyce J., Ryan R., Shepperd S., Thomas J. (2020). Synthesis without Meta-Analysis (SWiM) in Systematic Reviews: Reporting Guideline. BMJ.

[15] Ameli R., Sinaii N., West C.P., Luna M.J., Panahi S., Zoosman M., Rusch H.L., Berger A. (2020). Effect of a Brief Mindfulness-Based Program on Stress in Health Care Professionals at a US Biomedical Research Hospital: A Randomized Clinical Trial. JAMA Netw. Open.

[16] Wu Y., Ban Y., Pan G., Yao M., Liu L., Chen T., Wu H. (2025). Effects of Online Mindfulness-Based Stress Reduction Training on Depression and Anxiety Symptoms among Psychiatric Healthcare Workers in a Randomized Controlled Trial: The Mediating Role of Emotional Suppression. BMC Psychiatry.

[17] Liu X., Luo S., Wen X., Huang X., Wu J., Chen M., Jia P. (2025). The Effect of Mindfulness-Based Stress Reduction on Presenteeism among ICU Nurses: A Cluster Randomized Controlled Trial. PLoS ONE.

[18] Arts-de Jong M., Geurts D.E.M., Spinhoven P., Ruhé H.G., Speckens A.E.M. (2025). Mindfulness-Based Interventions for Mental Health Outcomes in Frontline Healthcare Workers During the COVID-19 Pandemic: A Randomized Controlled Trial. J. Gen. Intern. Med..

[19] Bhardwaj P., Pathania M., Bahurupi Y., Kanchibhotla D., Harsora P., Rathaur V.K. (2023). Efficacy of mHealth Aided 12-Week Meditation and Breath Intervention on Change in Burnout and Professional Quality of Life among Health Care Providers of a Tertiary Care Hospital in North India: A Randomized Waitlist-Controlled Trial. Front. Public Health.

[20] Çelik A.S., Kılınç T. (2022). The Effect of Laughter Yoga on Perceived Stress, Burnout, and Life Satisfaction in Nurses during the Pandemic: A Randomized Controlled Trial. Complement. Ther. Clin. Pract..

[21] Yıldırım D., Çiriş Yıldız C. (2022). The Effect of Mindfulness-Based Breathing and Music Therapy Practice on Nurses’ Stress, Work-Related Strain, and Psychological Well-Being During the COVID-19 Pandemic: A Randomized Controlled Trial. Holist. Nurs. Pract..

[22] Björkelund C., Petersson E.-L., Svenningsson I., Saxvik A., Wiegner L., Hensing G., Jonsdottir I.H., Larsson M., Wikberg C., Ariai N. (2024). Effects of Adding Early Cooperation and a Work-Place Dialogue Meeting to Primary Care Management for Sick-Listed Patients with Stress-Related Disorders: CO-WORK-CARE-Stress—A Pragmatic Cluster Randomised Controlled Trial. Scand. J. Prim. Health Care.

[23] Andani Y., Shatri H., Koesnoe S., Yunir E., Wiguna T., Wibowo H., Sawitri D.R., Sarwono S.J., Mansyur M., Ricardo W. (2025). Effects of Traditional Music Therapy on the Psycho-Neuro-Immuno-Endocrine Aspect of Burnout Syndrome in Healthcare Workers: A Randomized Controlled Trial. Narra J..

[24] Evans L.V., Bonz J.W., Buck S., Gerwin J.N., Bonner S., Ikejiani S., Moylan T., Joseph M., de Oliveira Almeida G., Ray J.M. (2025). An Adaptive Simulation Intervention Decreases Emergency Physician Physiologic Stress While Caring for Patients during COVID-19: A Randomized Clinical Trial. PLoS ONE.

[25] Chang Y.-C., Hsu M.-C., Ouyang W.-C. (2022). Effects of Integrated Workplace Violence Management Intervention on Occupational Coping Self-Efficacy, Goal Commitment, Attitudes, and Confidence in Emergency Department Nurses: A Cluster-Randomized Controlled Trial. Int. J. Environ. Res. Public Health.

[26] Fainstad T., Mann A., Suresh K., Shah P., Dieujuste N., Thurmon K., Jones C.D. (2022). Effect of a Novel Online Group-Coaching Program to Reduce Burnout in Female Resident Physicians: A Randomized Clinical Trial. JAMA Netw. Open.

[27] Saeidavi H., Nazari M., Ghahremani L., Nazari A. (2025). Self-Efficacy-Based Intervention for Stress Management in Health Centers Employees. BMC Psychol..

[28] Dincer B., Inangil D. (2021). The Effect of Emotional Freedom Techniques on Nurses’ Stress, Anxiety, and Burnout Levels during the COVID-19 Pandemic: A Randomized Controlled Trial. Explore.

[29] Antico L., Brewer J. (2025). Digital Mindfulness Training for Burnout Reduction in Physicians: Clinician-Driven Approach. JMIR Form. Res..

[30] López-Del-Hoyo Y., Fernández-Martínez S., Perez-Aranda A., Monreal-Bartolomé A., Barceló-Soler A., Camarero-Grados L., Armas-Landaeta C., Guzmán-Parra J., Carbonell V., Campos D. (2025). Effectiveness of a Web-Based Self-Guided Intervention (MINDxYOU) for Reducing Stress and Promoting Mental Health Among Health Professionals: Results From a Stepped-Wedge Cluster Randomized Trial. J. Med. Internet Res..

[31] Joshi S.P., Wong A.-K.I., Brucker A., Ardito T.A., Chow S.-C., Vaishnavi S., Lee P.J. (2022). Efficacy of Transcendental Meditation to Reduce Stress Among Health Care Workers: A Randomized Clinical Trial. JAMA Netw. Open.

[32] Zhang L., Huang S., Liu S., Huang Y., Chen S., Hu J., Xu M. (2024). Effectiveness of an Internet-Based Acceptance and Commitment Therapy Intervention for Reducing Psychological Distress in Health Care Professionals: Randomized Controlled Trial. J. Med. Internet Res..

[33] Abbasalizadeh M., Farsi Z., Sajadi S.A., Atashi A., Fournier A. (2024). The Effect of Resilience Training with mHealth Application Based on Micro-Learning Method on the Stress and Anxiety of Nurses Working in Intensive Care Units: A Randomized Controlled Trial. BMC Med. Educ..

[34] Prasad K., Poplau S., Brown R., Yale S., Grossman E., Varkey A.B., Williams E., Neprash H., Linzer M., Healthy Work Place (HWP) Investigators (2020). Time Pressure During Primary Care Office Visits: A Prospective Evaluation of Data from the Healthy Work Place Study. J. Gen. Intern. Med..

[35] Linzer M., Poplau S., Prasad K., Khullar D., Brown R., Varkey A., Yale S., Grossman E., Williams E., Sinsky C. (2019). Characteristics of Health Care Organizations Associated with Clinician Trust: Results from the Healthy Work Place Study. JAMA Netw. Open.

[36] Hersch R.K., Cook R.F., Deitz D.K., Kaplan S., Hughes D., Friesen M.A., Vezina M. (2016). Reducing Nurses’ Stress: A Randomized Controlled Trial of a Web-Based Stress Management Program for Nurses. Appl. Nurs. Res. ANR.

[37] Rossetti A., Loewy J., Chang-Lit W., van Dokkum N.H., Baumann E., Bouissou G., Mondanaro J., O’Connor T., Asch-Ortiz G., Mitaka H. (2023). Effects of Live Music on the Perception of Noise in the SICU/PICU: A Patient, Caregiver, and Medical Staff Environmental Study. Int. J. Environ. Res. Public Health.

[38] Gao Q., Yao Y., Wang R., Zhang X., Gudenkauf L.M., Xu G., Harrison S., Zheng L., Wang J., Chen G. (2024). Enhancing the Psychological Well-Being and Sleep Quality of Healthcare Providers with a Multimodal Psychological Support Program: A Randomized Controlled Trial. Front. Public Health.

[39] Mandal S., Misra P., Sharma G., Sagar R., Kant S., Dwivedi S.N., Lakshmy R., Goswami K. (2021). Effect of Structured Yoga Program on Stress and Professional Quality of Life Among Nursing Staff in a Tertiary Care Hospital of Delhi-A Small Scale Phase-II Trial. J. Evid.-Based Integr. Med..

[40] Pieper C., Lausen M., Kröckert D., Klemp Y., Baer U. (2025). Creative Strengthening Groups as a Potential Intervention to Enhance Job Satisfaction and Reduce Levels of Burnout in Healthcare Professionals: Results from the Randomized Controlled Trial UPGRADE. BMC Health Serv. Res..

[41] Degen L., Linden K., Seifried-Dübon T., Werners B., Grot M., Rind E., Pieper C., Eilerts A.-L., Schroeder V., Kasten S. (2021). Job Satisfaction and Chronic Stress of General Practitioners and Their Teams: Baseline Data of a Cluster-Randomised Trial (IMPROVEjob). Int. J. Environ. Res. Public Health.

[42] Teo I., Chay J., Cheung Y.B., Sung S.C., Tewani K.G., Yeo L.F., Yang G.M., Pan F.T., Ng J.Y., Abu Bakar Aloweni F. (2021). Healthcare Worker Stress, Anxiety and Burnout during the COVID-19 Pandemic in Singapore: A 6-Month Multi-Centre Prospective Study. PLoS ONE.

[43] Mohebbi Z., Dehkordi S.F., Sharif F., Banitalebi E. (2019). The Effect of Aerobic Exercise on Occupational Stress of Female Nurses: A Controlled Clinical Trial. Investig. Educ. Enferm..

[44] Imamura K., Tran T.T.T., Nguyen H.T., Sasaki N., Kuribayashi K., Sakuraya A., Bui T.M., Nguyen A.Q., Nguyen Q.T., Nguyen N.T. (2021). Effect of Smartphone-Based Stress Management Programs on Depression and Anxiety of Hospital Nurses in Vietnam: A Three-Arm Randomized Controlled Trial. Sci. Rep..

[45] Xue M., Yu P., Gu Z., Sun Y. (2025). Online Narrative Therapy Intervention Improves Post-Traumatic Stress Disorder Symptoms, Perceived Stress, Anxiety, and Depression in Nurses: A Randomized Controlled Trial. Rev. Bras. Psiquiatr..

[46] Ho A.H.Y., Tan-Ho G., Ngo T.A., Ong G., Chong P.H., Dignadice D., Potash J. (2019). A Novel Mindful-Compassion Art Therapy (MCAT) for Reducing Burnout and Promoting Resilience for End-of-Life Care Professionals: A Waitlist RCT Protocol. Trials.

[47] Ahmed Pihlgren S., Johansson L., Holmes E.A., Kanstrup M. (2024). Exploring Healthcare Workers’ Experiences of a Simple Intervention to Reduce Their Intrusive Memories of Psychological Trauma: An Interpretative Phenomenological Analysis. Eur. J. Psychotraumatol..

[48] Bernburg M., Vitzthum K., Groneberg D.A., Mache S. (2016). Physicians’ Occupational Stress, Depressive Symptoms and Work Ability in Relation to Their Working Environment: A Cross-Sectional Study of Differences among Medical Residents with Various Specialties Working in German Hospitals. BMJ Open.

[49] Agarwal A.K., Southwick L., Gonzales R.E., Bellini L.M., Asch D.A., Shea J.A., Mitra N., Yang L., Josephs M., Kopinksy M. (2024). Digital Engagement Strategy and Health Care Worker Mental Health: A Randomized Clinical Trial. JAMA Netw. Open.

[50] Sexton J.B., Adair K.C., Cui X., Tawfik D.S., Profit J. (2022). Effectiveness of a Bite-Sized Web-Based Intervention to Improve Healthcare Worker Wellbeing: A Randomized Clinical Trial of WISER. Front. Public Health.

[51] Sexton J.B., Adair K.C. (2024). Well-Being Outcomes of Health Care Workers After a 5-Hour Continuing Education Intervention: The WELL-B Randomized Clinical Trial. JAMA Netw. Open.

[52] Coelhoso C.C., Tobo P.R., Lacerda S.S., Lima A.H., Barrichello C.R.C., Amaro E., Kozasa E.H. (2019). A New Mental Health Mobile App for Well-Being and Stress Reduction in Working Women: Randomized Controlled Trial. J. Med. Internet Res..

[53] Timmins G., Williamson S., Cassells A., Davis K., Dong L., Tobin J.N., Gidengil C., Meredith L.S., Chen P.G. (2025). Health Care Worker Experiences with a Brief Peer Support and Well-Being Intervention during the COVID-19 Pandemic. BMC Health Serv. Res..

[54] Srikasem S., Seephom S., Viriyopase A., Phutrakool P., Khowinthaseth S., Narajeenron K., ER-VIPE Study Group (2025). Comparing the Effectiveness of Multimodal Learning Using Computer-Based and Immersive Virtual Reality Simulation-Based Interprofessional Education with Co-Debriefing, Medical Movies, and Massive Online Open Courses for Mitigating Stress and Long-Term Burnout in Medical Training: Quasi-Experimental Study. JMIR Med. Educ..

[55] Belkić K. (2025). Toward Better Prevention of Physician Burnout: Insights from Individual Participant Data Using the MD-Specific Occupational Stressor Index and Organizational Interventions. Front. Public Health.

[56] Janicki A.J., Frisch S.O., Patterson P.D., Brown A., Frisch A. (2020). Emergency Medicine Residents Experience Acute Stress While Working in the Emergency Department. West. J. Emerg. Med..

[57] Mulfinger N., Jarczok M.N., Müller A., Genrich-Hasken M., Worringer B., Küllenberg J.K., Junne F., Rapp F., Rieger M.A., Rothermund-Nassir E. (2025). Effectiveness of a Multilevel Intervention to Improve Mental Health of Hospital Workers: The SEEGEN Multicenter Cluster Randomized Controlled Trial. PLoS ONE.

[58] Benzo R.P., Kirsch J.L., Nelson C. (2017). Compassion, Mindfulness, and the Happiness of Healthcare Workers. Explore.

[59] Pollock A., Campbell P., Cheyne J., Cowie J., Davis B., McCallum J., McGill K., Elders A., Hagen S., McClurg D. (2020). Interventions to Support the Resilience and Mental Health of Frontline Health and Social Care Professionals during and after a Disease Outbreak, Epidemic or Pandemic: A Mixed Methods Systematic Review. Cochrane Database Syst. Rev..

[60] Bock L., Westemeyer L., Moschner N., Rana M., Rana M. (2025). Resilience Among Healthcare Staff: A Randomized Controlled Trial of a Digital Training Program. J. Clin. Psychol. Med. Settings.

[61] Duva I.M., Higgins M.K., Baird M., Lawson D., Murphy J.R., Grabbe L. (2022). Practical Resiliency Training for Healthcare Workers during COVID-19: Results from a Randomised Controlled Trial Testing the Community Resiliency Model for Well-Being Support. BMJ Open Qual..

[62] Hsieh H.-F., Huang I.-C., Liu Y., Chen W.-L., Lee Y.-W., Hsu H.-T. (2020). The Effects of Biofeedback Training and Smartphone-Delivered Biofeedback Training on Resilience, Occupational Stress, and Depressive Symptoms among Abused Psychiatric Nurses. Int. J. Environ. Res. Public Health.

[63] Lee D., Lee W.J., Choi S.-H., Jang J.-H., Kang D.-H. (2020). Long-Term Beneficial Effects of an Online Mind-Body Training Program on Stress and Psychological Outcomes in Female Healthcare Providers: A Non-Randomized Controlled Study. Medicine.

[64] Mediavilla R., Felez-Nobrega M., McGreevy K.R., Monistrol-Mula A., Bravo-Ortiz M.-F., Bayón C., Giné-Vázquez I., Villaescusa R., Muñoz-Sanjosé A., Aguilar-Ortiz S. (2023). Effectiveness of a Mental Health Stepped-Care Programme for Healthcare Workers with Psychological Distress in Crisis Settings: A Multicentre Randomised Controlled Trial. BMJ Ment. Health.

[65] Margareta B., Ulf I., C F.G. (2025). Effects of Ethics Communication in Health Care: A Cluster Randomised Controlled Trial. BMC Med. Ethics.

[66] Azizi T.H., Nayeri N.D., Jackson A.C., Bahramnezhad F. (2026). Investigating the Impact of the RENEW Program on Enhancing Nurses’ Resilience: A Randomized Controlled Trial. BMC Psychol..

[67] Super A., Yarker J., Lewis R., Keightley S., Summers D., Munir F. (2024). Developing Self-Compassion in Healthcare Professionals Utilising a Brief Online Intervention: A Randomised Waitlist Control Trial. Int. J. Environ. Res. Public Health.

[68] Imai R., Konishi T., Mibu A., Tanaka K., Nishigami T. (2021). Effect of Pain Neuroscience Education and Exercise on Presenteeism and Pain Intensity in Health Care Workers: A Randomized Controlled Trial. J. Occup. Health.

